# Characteristics of late presenters in Bucharest

**DOI:** 10.7448/IAS.17.4.19691

**Published:** 2014-11-02

**Authors:** Raluca Elena Jipa, Eliza Manea, Serban Benea, Iulia Niculescu, Otilia Benea, Mariana Mardarescu, Cosmina Andrei, Ruxandra Moroti, Adriana Hristea

**Affiliations:** 1 Clinical Department III, National Institute of Infectious Diseases “Prof. Dr. Matei Bals”, Bucharest, Romania; 2 Clinical Department V, National Institute of Infectious Diseases “Prof. Dr. Matei Bals”, Bucharest, Romania; 3 Clinical Department VIII, National Institute of Infectious Diseases “Prof. Dr. Matei Bals”, Bucharest, Romania

## Abstract

**Introduction:**

Late presentation is associated with increased healthcare costs, rates of HIV transmission and poor outcome. In Romania, in 2012, one third of individuals with new HIV diagnosis were late presenters (LP).

**Objective:**

The aim of the study was to evaluate the epidemiological and clinical characteristics associated with late presentation.

**Methods:**

We retrospectively studied patients over 18 years old, notified in our institution between January 2012 and December 2013, including 499 out of 727 newly diagnosed patients in Bucharest. LP were defined as patients presenting with CD4 T-cell count below 350 cells/mm^3^ or with an AIDS defining event. Patients with advanced HIV disease (AHD) were defined as persons with a CD4 T-cell count below 200 cells/mm^3^. Differences between groups were analyzed using the Mann-Whitney U test for continuous variables and the chi-square test for dichotomous variables. Multivariable analysis was performed using binary logistic regression.

**Results:**

Out of 499 patients included, 362 (72%) were male. The median age was 30 (IQR 26–36). A total of 302 (61%) were LP and 184 (37%) were patients with AHD. A total of 170 (34%) were asymptomatic and 114 (23%) presented with an AIDS-defining event. The median CD4 count was 293 cells/mm^3^ (IQR 125–471) and the median HIV viral load was 100,191 copies/mL (IQR 34,560–272,936). Characteristics of LP compared with non-LP are shown in Table 1. Stage C disease has been shown by multivariable analysis to be associated with LP (p<0.001, OR=11.56, 95% CI 4.94–27.03).

**Conclusions:**

More than half of newly HIV diagnosed patients in Bucharest were LP. The proportion of LP was highest among heterosexually acquired cases. Although most our patients were young, late presentation was associated with age over 35 years. The lower proportion of LP among IVDU compared with those heterosexually infected could be explained by a higher proportion of HIV screening tests among IVDU.

**Table 1:** Epidemiological, clinical and laboratory characteristics in late presenters versus non-late presenters

	Late presenters, N=302	Non-late presenters, N=197	p value; OR [95% CI]
Male, N (%)	214 (71)	148 (75)	0.307; 1.2 [0.8–1.8]
Age (years) (median, IQR)	32 (27–38)	29 (25–34)	<0.001
Age≥35 years, N (%)	105 (35)	38 (19)	<0.001; 2.2 [1.4–3.4]
Level of education-under 8th grade, N (%)	146 (48)	122 (62)	0.003; 0.5 [0.3–0.8]
Transmission mode: IVDU, N (%)	124 (41)	111 (56)	0.001; 0.5 [0.3–0.7]
Transmission mode: Heterosexual, N (%)	149 (49)	68 (35)	0.001; 1.8 [1.2–2.6]
Transmission mode: MSM, N (%)	28 (9)	20 (10)	0.174; 1.6 [0.8–3]
Stage A, N (%)	107 (35)	141 (72)	<0.001; 0.2 [0.1–0.3]
Stage B, N (%)	88 (29)	49 (25)	0.307; 0.8 [0.5–1.2]
Stage C, N (%)	107 (35)	7 (4)	<0.001; 14.8 [6.7–32.8]
HIV-RNA≥100,000 copies/mL, N (%)	199 (66)	103 (52)	0.003; 1.7 [1.2–2.5]
Chronic hepatitis C, N (%)	129 (43)	118 (60)	<0.001; 0.5 [0.3–0.7]
Chronic hepatitis B, N (%)	38 (13)	22 (11)	0.67; 1.1 [0.6–2]
Mortality, N (%)	40 (13)	6 (3)	<0.001; 4.8 [2.1–12.8]

IQR=Interquartile range; IVDU=Intravenous drug users; MSM=men having sex with men.

